# Intramuscular preadipocytes impede differentiation and promote lipid deposition of muscle satellite cells in chickens

**DOI:** 10.1186/s12864-018-5209-5

**Published:** 2018-11-26

**Authors:** Liping Guo, Huanxian Cui, Guiping Zhao, Ranran Liu, Qinghe Li, Maiqing Zheng, Yuming Guo, Jie Wen

**Affiliations:** 1 Institute of Animal Sciences, Chinese Academy of Agricultural Sciences, Beijing, 100193 China; 2 College of Animal Science and Technology, China Agricultural University, Beijing, 100193 China; 3 State Key Laboratory of Animal Nutrition, Beijing, 100193 China

**Keywords:** Muscle satellite cells, Intramuscular preadipocytes, Interaction, Myogenic differentiation, Lipid deposition, Chicken

## Abstract

**Background:**

Skeletal muscle satellite cells (MSC) are crucial for postnatal growth and regeneration of skeletal muscle. An interaction exists between MSC and intramuscular preadipocytes (IMPA). This study is the first to investigate the effects of IMPA on MSC in chickens and unveil the molecular mechanisms by transcriptome analysis.

**Results:**

Primary MSC and IMPA were isolated from the pectoralis major muscle of 7-day-old chickens. After both cell types reached confluence, MSC were cultured alone or co-cultured with IMPA for 2 or 4 d. MSC treated for 2 d were subjected to RNA-seq. A total of 1653 known differentially expressed genes (DEG) were identified between co-cultured and mono-cultured MSC (|log2 FC| ≥ 1, FDR < 0.01). Based on Gene Ontology analysis, 48 DEG related to muscle development were screened, including the key genes *MYOD1*, *MYOG*, *PAX7*, and *TMEM8C*. The 44 DEG related to lipid deposition included the key genes *CD36, FABP4, ACSBG2, CYP7A1* and *PLIN2*. Most of the DEG related to muscle development were downregulated in co-cultured MSC, and DEG related to lipid deposition were upregulated. Immunofluorescence of MHC supported IMPA impeding differentiation of MSC, and Oil Red O staining showed concurrent promotion of lipid deposition. Pathway analysis found that several key genes were enriched in JNK/MAPK and PPAR signaling, which may be the key pathways regulating differentiation and lipid deposition in MSC. Additionally, pathways related to cell junctions may also contribute to the effect of IMPA on MSC.

**Conclusions:**

The present study showed that IMPA impeded differentiation of MSC while promoting their lipid deposition. Pathway analysis indicated that IMPA might inhibit differentiation via the JNK/MAPK pathway, and promote lipid deposition via the PPAR pathway. This study supplies insights into the effect of IMPA on MSC, providing new clues on exposing the molecular mechanisms underlying the interplay between skeletal muscle and intramuscular fat in chickens.

**Electronic supplementary material:**

The online version of this article (10.1186/s12864-018-5209-5) contains supplementary material, which is available to authorized users.

## Background

Chickens are important farm animals throughout the world, producing eggs and high quality meat for humans. Skeletal muscle mass and intramuscular fat content are both important traits for meat-producing chickens but attaining an appropriate muscle and fat ratio, reflecting the balance between muscle and adipose cells, is a great challenge for the broiler industry [1]. Muscle satellite cells (MSC) are skeletal muscle-specific stem cells, located between the basement membrane and sarcolemma of muscle fibers [2–4]. Through their proliferation and subsequent fusion with existing myofibers, MSC provide new myonuclei to the muscle fibers, making them crucial to postnatal growth and regeneration of skeletal muscle [5–7]. Intramuscular preadipocytes (IMPA) differentiate into mature adipocytes within bundles of muscle fibers, thereby contributing to flavor and shear characteristics, thus improving meat quality [8–10].

Intercellular communication is a fundamental process in biology, and an interaction exists between MSC and IMPA. Bovine preadipocytes increase expression of adipogenic genes in bovine satellite cells [11]. Satellite cell-derived myofibers of mice exerted a strongly inhibitory effect on adipogenesis of mesenchymal progenitors [12]. Similar results were obtained in porcine satellite cells that inhibited the differentiation of preadipocytes [13]. Both MSC and IMPA are derived from a common pool of mesenchymal stem cells and are located adjacent to each other; it is reasonable to suppose that there is intercellular communication between the two. There has been no study of the interactions between IMPA and MSC in chickens.

Co-culture of different cell types, separated by a permeable membrane, permits investigating interactions between distinct cell types, as it can mimic the in vivo cellular environment [14]. Medium constituents, including signaling molecules, exchange freely, thus effectuating cell-cell communication while preventing physical contact and admixing of the different cell populations enabling harvesting of the cells for subsequent analyses.

The present study has used a co-culture system to investigate the influence of IMPA on MSC in young chickens; transcriptome analysis was used to explore the underlying molecular mechanisms.

## Methods

### Isolation of chicken muscle satellite cells and intramuscular preadipocytes

The 7-day-old Beijing-You chickens were obtained from the experimental farm of the IAS (CAAS, Beijing, China). Primary cells were isolated as described previously with a slightly modification [15]. Chickens were stunned by electrical stunning (120 mA, 50 Hz) and then slaughtered with a quick, single cut to the throat. After sprayed with 75% ethanol, the pectoralis major muscle was isolated. The muscle was minced to 1 mm^3^ segments and digested for 60 min with 0.1% *w*/*v* type I collagenase (Gibco, Grand Island, NY). After terminating digestion with complete medium consisting of Dulbecco’s modified Eagle’s medium (DMEM)/F12 (Gibco), 10% fetal bovine serum (FBS, Gibco), and 1% penicillin/streptomycin (Gibco), the cell suspension was centrifuged at 350 g for 10 min. The top layer containing mature adipocytes and the bottom pellet containing MSC were separately collected. The mature adipocyte layer was inoculated into a 25-cm^2^ cell culture flask containing complete medium. The flask was incubated inverted for 6 d enabling adipocytes to attach to the upper surface and dedifferentiate, and subsequently re-inverted for another 6 d to allow the adherent cells to proliferate.

The cell pellet, containing MSC, was resuspended in complete medium, as above, filtered through 100- and 40-μm sterile sieves, and then cultured in complete medium in 100-mm dishes. After cells were incubated for 2 h, the medium containing unattached cells was transferred to a new dish, and purified MSC were obtained.

All cells were incubated in a humidified atmosphere of 5% CO2 at 37 °C. After reaching 80% confluence, both cell types were passaged with 0.25% trypsin-EDTA (Gibco), and passage 3 cells were used for further experiments.

### Co-culture of muscle satellite cells and intramuscular preadipocytes

Cells were co-cultured using Transwell plates with 0.4-μm membrane inserts (Corning Inc., Kennebunk, ME). The MSC (2 × 10^5^ per well) were plated in the lower wells of the 6-well plates, and IMPA (1 × 10^5^ per well) were seeded into the upper inserts, initially in separate plates, i.e., not in a co-culture configuration. After both cell types reached confluence, the upper inserts containing IMPA were then transferred to the lower wells containing MSC to establish the co-cultures. The mono-cultured MSC served as controls. Following co-culture or mono-culture for 2 or 4 d, MSC were harvested for further analysis. Comparisons between the two culture arrangements were made with 3 repetitions to allow for statistical evaluation.

### Immunofluorescence

Myosin heavy chain (MHC) was used as a marker of MSC differentiation. After washing 3 times with PBS, the MSC were fixed in 4% paraformaldehyde for 20 min, permeabilized in 0.25% Triton X-100 for 15 min, and then blocked with 10% goat serum (CWBio, Beijing, China) for 30 min. Subsequently, the cells were incubated with primary anti-MHC hybridoma supernatant (1:100, Developmental Studies Hybridoma Bank, Iowa City, IA) overnight at 4 °C. After thorough washing, the cells were incubated in the dark for 1 h with fluorescein isothiocyanate (FITC)-conjugated goat anti-mouse IgG (1:100, CWBio, Beijing, China). Fluorescence signals of MSC in the lower chambers were detected and photographed by confocal fluorescence microscopy (Nikon TE-2000-E, Tokyo, Japan) [16].

### Oil red O staining

After 4 d of co-culture or mono-culture, the MSC were washed 3 times with PBS, fixed in 4% paraformaldehyde for 15 min, and then stained with a 0.5% solution of Oil Red O in 60% isopropanol for 1 h at room temperature. The cells were washed 3 times with PBS, and the stained fat droplets in the MSC were visualized by light microscopy and photographed [17]. Isopropanol (100%) was incubated for 10 min to extract Oil Red O from the cells and then the extracts were transferred to a 96-well-plate. The OD_510_ was measured photometrically with a microplate reader (Molecular Devices, SpectraMax M2, San Jose, CA) [18].

### RNA isolation and sequencing

After co-culture or mono-culture for 2 d, MSC were lysed by TRIzol reagent (Invitrogen Corp., Carlsbad, CA) and total RNA was extracted according to the manufacturer’ protocol. The RNA concentration and integrity were assessed using a 2100 Bioanalyzer and RNA 6000 Nano Kit (Agilent Technologies, Santa Clara, CA) separately. RNA samples with an A260/A280 ratio between 1.8 and 2.0 and a RNA Integrity Number > 8.0 were used for RNA sequencing and quantitative real-time PCR (qPCR). Based on ultra-high throughput sequencing (HiSeq 2500; Illumina, San Diego, CA), RNA-sequencing was performed by Annoroad Genomics (Beijing, China).

### Bioinformatics analysis of RNA-seq

Sequence adapters and low quality reads (read quality < 30) were removed using Trimmomatic (v0.32) [19]. Quality control checks on raw sequence data were performed with FastQC. Then, sequencing reads were mapped to the chicken reference genome [Ensembl Galgal4 (GCA_000002315.2)] using the HISAT(v2.0.4) program. To calculate the expression quantity of each transcript, alignment results were analyzed by the Cufflinks (v2.0.2) program. The FPKM (fragments per kilobase of transcript per million mapped fragments) method was used to quantify gene expression. Analyses of differential expression of transcripts were performed with edgeR (v2.2.5). Genes with a FDR value of less than 1% and fold-change ≥2 were considered to be differentially expressed genes (DEG).

Principal component analysis (PCA) was performed to identify the variability and repeatability of samples. A volcano plot was used to visualize the overall distribution of differentially expressed genes. Gene ontology (GO) analysis of DEG was performed using DAVID functional annotation clustering [20]. Kyoto Encyclopedia of Genes and Genomes (KEGG) pathway enrichment analysis was performed by KOBAS 3.0 (http://kobas.cbi.pku.edu.cn). Cytoscape was used to depict a pathway network of enriched pathways.

The significance level for GO terms and the KEGG pathway was set with the *P*-value < 0.05.

### Quantitative real-time PCR

The specific primers (Additional file 1: Table S1) were designed using Primer Premier 6.0 software, and were subsequently synthesized by the Sangon Biotech Corp (Shanghai, China). After reverse transcription, qPCR was performed using a SYBR Fast qPCR Master Mix (KAPA, Wilmington, MA).

The qPCR mixture contained 10 μL of 2× iQ™ SYBR Green Supermix, 0.5 μL (10 mM) of each primer and 20 ng of cDNA, with ddH_2_O added to 20 μL. Samples were amplified using the real-time PCR Detection System ABI 7500 (Applied Biosystems, Shanghai, China). The PCR cycle parameters were as follows: 95 °C for 3 min, then 40 cycles of 95 °C for 3 s, and 60 °C for 34 s. The amplification procedure was performed with 3 repetitions for each sample. The 2^-ΔΔCt^ method was used to calculate the relative abundance of transcripts [21].

### Statistical analysis

All data are presented as the mean ± standard deviation (SD), for 3 replicates from each experiment. Statistically significant differences between the 2 culture conditions were tested by independent-samples *t*-tests using SAS 9.2 software. *P* < 0.05 (*) or *P* < 0.01 (**) was considered to be significant. All figures were constructed using GraphPad Prism version 5.02 (GraphPad Software Inc., La Jolla, CA).

## Results

### Differential expression analysis of mono-cultured and co-cultured MSC

**Fig. 1:**
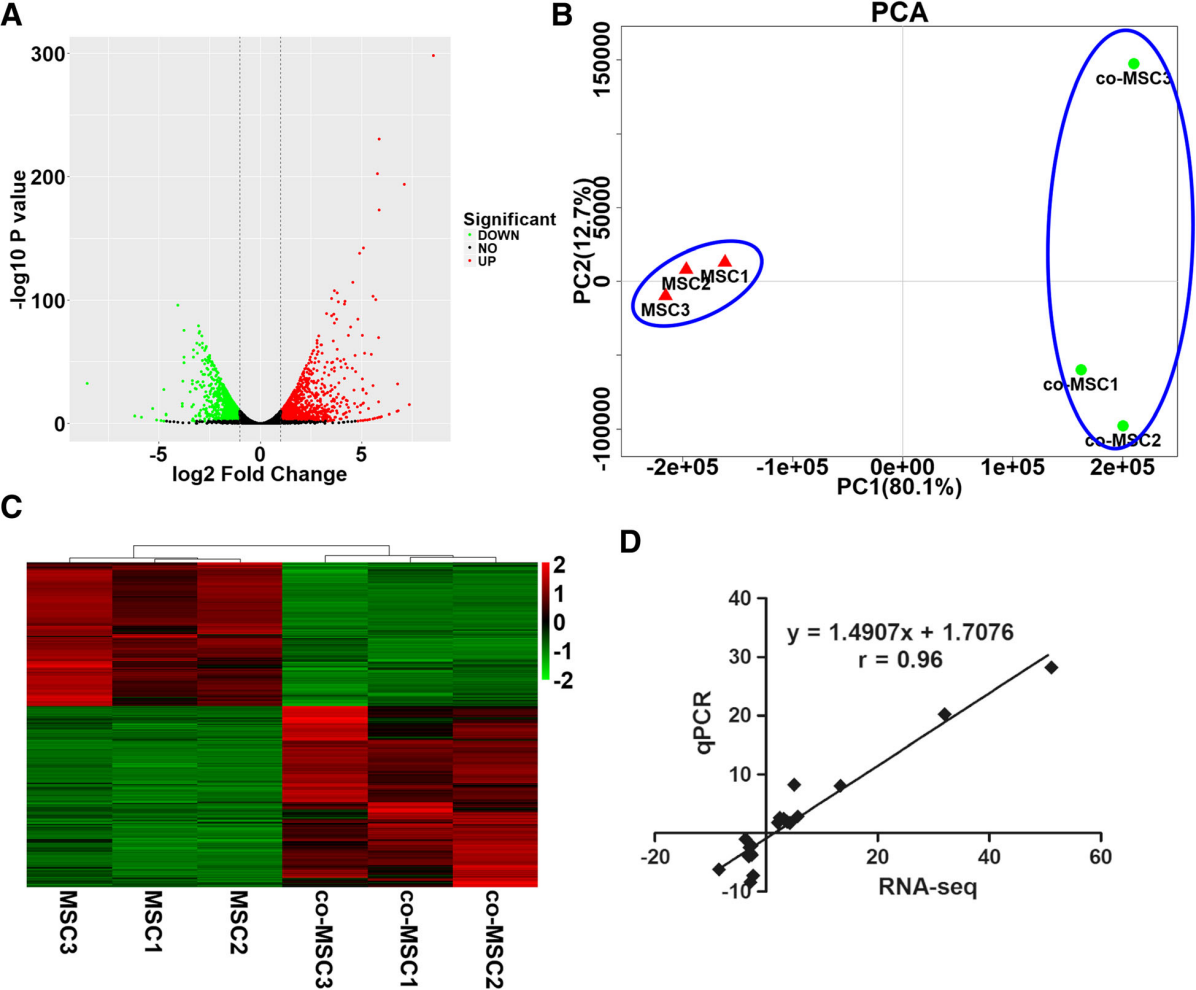
RNA-seq analysis and verification. **a** Volcano plot. The red dots represent significantly upregulated genes, the green dots represent significantly downregulated genes (|log2 FC| ≥ 1 and FDR < 0.01), and the black dots represent insignificant differentially expressed genes. **b** Principal component analysis (PCA) of the RNA-seq data based on the gene transcription of 6 independent samples. The co-cultured and mono-cultured MSC were generally distinctly clustered within the first principal component, explaining 80% of the observed variation. **c** Hierarchical clustering. Hierarchical clustering analyses were performed based on all DEG, and the co-cultured and mono-cultured MSC were clustered. **d** Correlation analysis of the relative expression levels of 20 DEG between the RNA-seq and qPCR

A total of 1653 known DEG, of which 922 were upregulated and 731 were downregulated, were identified in MSC that were co-cultured in the presence of IMPA (|log2 FC| ≥ 1 and FDR < 0.01) (Fig. 1a and Additional file 2: Table S2).

To evaluate the consistency and variance of the 6 samples, PCA (based on all gene transcripts) and hierarchical clustering (based on all DEG) were performed. PCA demonstrated that the co-cultured and mono-cultured MSC were distinctly clustered (Fig 1b), and this was supported by the hierarchical clustering result (Fig. 1c), verifying acceptable repeatability in each and obvious difference between the culture conditions.

Accuracy of the RNA-seq data was examined by qPCR of 20 randomly chosen DEG and correlation between the 2 methods. Fold changes of the DEG were significantly correlated over a wide range (*r* = 0.96, *P* < 0.01) (Fig. 1d), confirming reliability of the RNA-seq results.

### Functional enrichment analysis of the DEG

The function of the known DEG was examined by GO enrichment analysis. A total of 75 Biological Process (BP) terms were significantly enriched (*P* < 0.05) (Additional file 3: Table S3). These BP terms were mainly associated with muscle development, lipid metabolism, ossification, angiogenesis, cell activities (cell growth, adhesion, migration, and differentiation), regulation of ion transmembrane transport, protein phosphorylation, and signal transduction (*P* < 0.05). The DEG related to muscle development and lipid deposition were examined further. There were 48 DEG related to muscle differentiation and development, including the key genes *MYOD1*, *MYOG*, *PAX7*, and *TMEM8C* (Additional file 4: Table S4). The 44 DEG related to lipid deposition included the key genes *CD36*, *FABP4*, *ACSBG2*, *CYP7A1* and *PLIN2* (Additional file 5: Table S5).

**Fig. 2:**
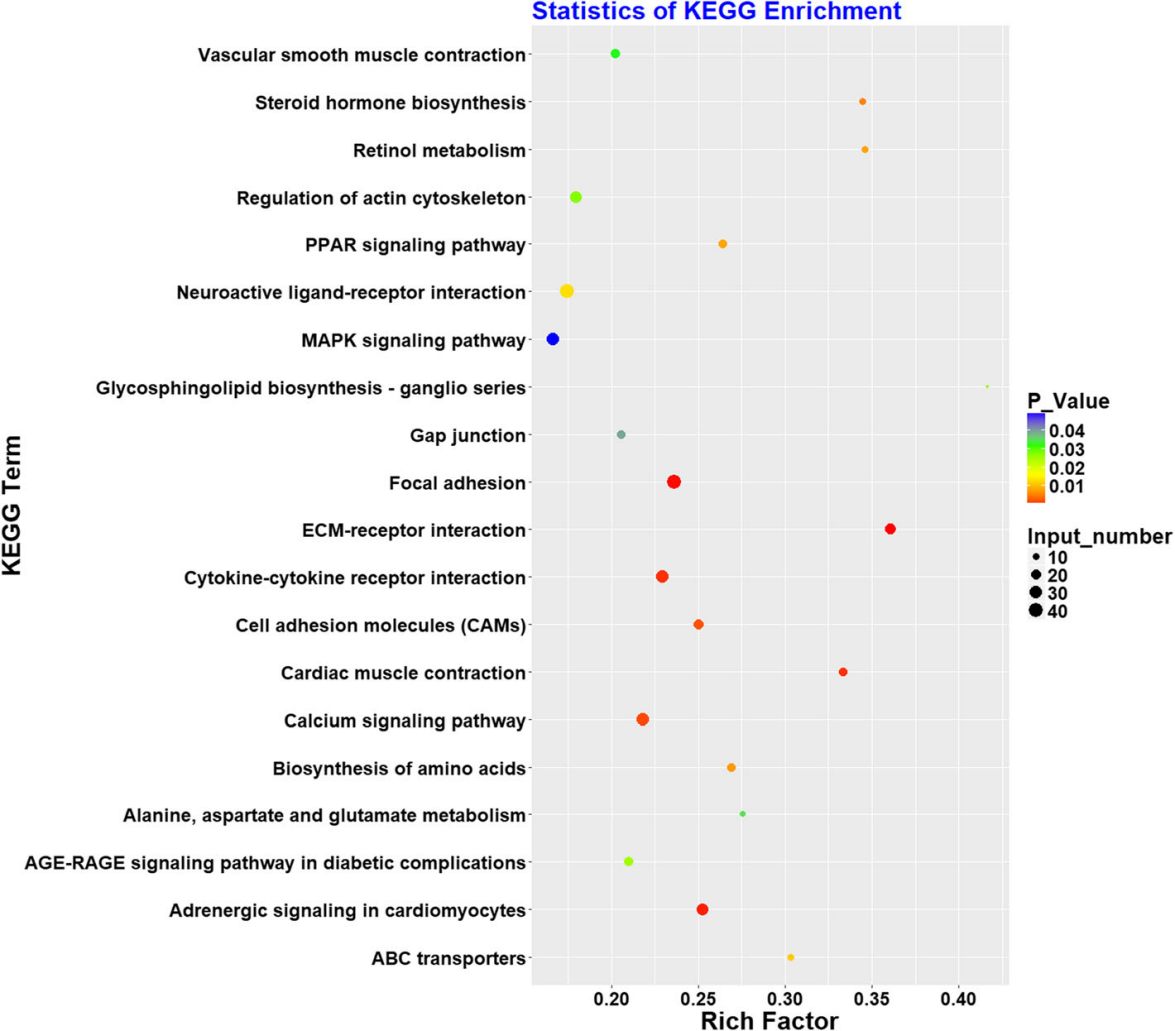
KEGG pathway analysis of DEG. Advanced bubble chart shows enrichment of differentially expressed genes in signaling pathways. The *x*-axis represents rich factor (rich factor = number of DEG enriched in the pathway/number of all genes in the background gene set). The *y*-axis represents the enriched pathway. Color represents enrichment significance, and size of the bubble represents the number of DEG enriched in the pathway

After KEGG pathway analysis, 20 pathways were significantly enriched (Fig. 2 and Additional file 6: Table S6). Consistent with the GO results, several of these pathways were related to muscle development and contraction, including MAPK signaling, Adrenergic signaling in cardiomyocytes, Cardiac muscle contraction, Vascular smooth muscle contraction, and Calcium signaling pathway. Many of the pathways were involved in lipid deposition, including PPAR signaling, Glycosphingolipid biosynthesis, and ABC transporters. In addition, six enriched pathways were related to cell junction, including extracellular matrix (ECM)-receptor interaction, Focal adhesion, Cell adhesion molecules (CAMs), Gap junction, Cytokine-cytokine receptor interaction, and Regulation of actin cytoskeleton.

### IMPA constrain differentiation of MSC

Of the 48 DEG related to muscle development, 15 DEG were related to skeletal muscle cell differentiation, 12 DEG were related to myofibril and myosin, 14 DEG were related to muscle organ development and 7 DEG were related to muscle contraction (Table 1). The majority (83.3%) of DEG related to muscle development were significantly downregulated in MSC that were co-cultured, indicating that differentiation of MSC was reduced by the influence of IMPA.

**Table 1:** DEG related to muscle differentiation and development

Terms	Gene name	Fold change	Terms	Gene name	Fold change
muscle organ development (13/14)	*SGCD*	0.46	skeletal muscle cell differentiation (10/15)	*MYOG*	0.33
	*CRYAB*	0.32		*MYOD1*	0.39
	*JPH1*	0.19		*ANKRD1*	0.19
	*SGCG*	0.35		*ASB2*	0.24
	*ALX4*	0.48		*SMYD1*	0.29
	*TNNT2*	0.50		*ACTA1*	0.44
	*TNNC1*	0.42		*FAM65B*	0.37
	*TPM1*	0.40		*HOMER1*	0.39
	*ACTA2*	0.38		*TMEM8C*	0.28
	*ACTC1*	0.42		*NTN3*	0.26
	*TAGLN*	0.44		*HEYL*	3.82
	*PVALB*	0.39		*BTG2*	2.94
	*CASQ2*	0.37		*GPX1*	2.75
	*MB*	2.73		*CXCL14*	4.23
				*HOPX*	2.82
myofibril and myosin (11/12)	*MYL1*	0.30			
	*CALD1*	0.46	muscle contraction (6/7)	*SYNM*	0.43
	*MYL4*	0.45		*LMOD2*	0.29
	*MYH13*	0.13		*STAC*	0.16
	*MYH10*	0.44		*ACTN2*	0.39
	*MYO5A*	0.48		*MYOM2*	0.31
	*MYO1H*	0.48		*LMOD1*	0.41
	*MYO16*	0.13		*CLCN1*	2.90
	*MYH1E*	0.28			
	*MYH15*	0.28			
	*CFL2*	0.50			
	*MYO1F*	2.84			

The first number in the parentheses represents the downregulated genes, and the second represents the total genes involved in the term

**Fig. 3:**
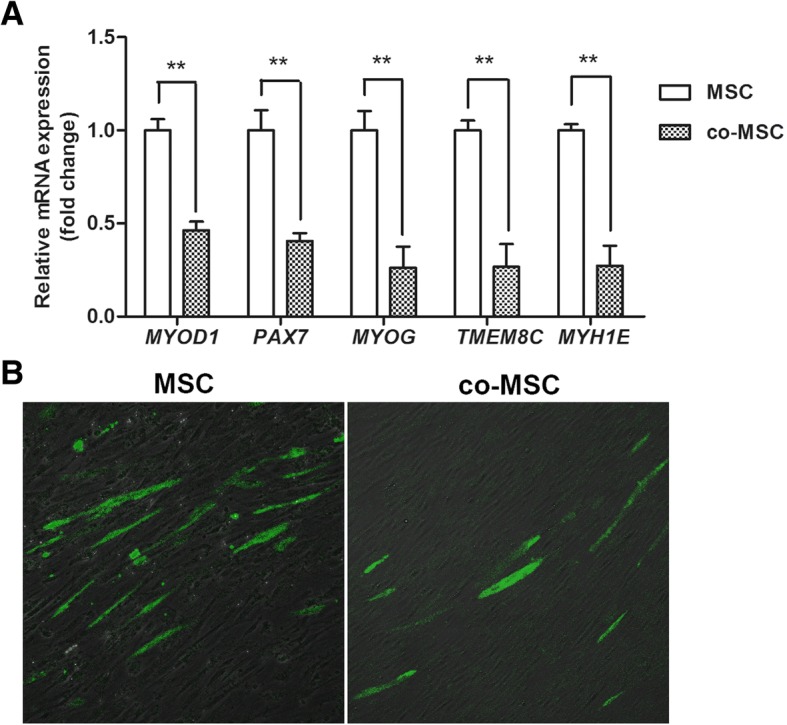
Myogenic differentiation of MSC is reduced by co-culture with IMPA. **a** qPCR assays of transcripts of 5 key genes (*MYOD1*, *PAX7*, *MYOG*, *TMEM8C* and *MYHIE*) related to myogenic differentiation in co-cultured or mono-cultured MSC on day 2. The key genes related to myogenic differentiation were all downregulated in co-cultured MSC. The data are expressed as the mean ± SD (** *P* < 0.01). **b** Immunofluorescent staining of myosin heavy chain (MHC) in co-cultured or mono-cultured MSC on day 2 indicating fewer MHC-positive cells in co-cultured MSC

To explore this negative influence of IMPA on MSC differentiation, transcript abundance of 5 key genes involved in muscle differentiation was quantified by qPCR. In addition, immunofluorescence was used to explore MHC (the major marker of terminal myogenic differentiation, encoded by *MYH1E*) to directly assess differentiation of MSC. Compared with mono-cultured MSC, the abundance of *MYOD1*, *MYH1E*, *PAX7*, *TMEM8C*, and *MYOG* transcripts was significantly downregulated in co-cultured MSC on day 2 (Fig. 3a). Immunofluorescence results showed that MHC-positive cells were also reduced in number in co-cultured MSC on day 2 (Fig. 3b). These results confirmed that IMPA suppressed differentiation of the MSC.

### IMPA promote lipid accumulation in MSC

Of the 44 DEG related to lipid deposition, 19 DEG related to fatty acid binding and transport, 13 related to fat cell differentiation, and 12 related to cholesterol metabolism (Table 2). The majority (81.8%) of DEG related to lipid deposition were significantly upregulated in co-cultured MSC, indicating that IMPA promoted lipid deposition in the MSC.

**Table 2:** DEG related to lipid deposition

Terms	Gene name	Fold change	Terms	Gene name	Fold change
fatty acid binding and transport (16/19)	*APOD*	6.30	fat cell differentiation (10/13)	*HTR2C*	2.56
	*FABP4*	13.75		*LAMA4*	11.81
	*FABP5*	3.95		*MB*	2.73
	*LCN8*	372.31		*RGS2*	12.18
	*PLIN2*	4.55		*SFRP2*	2.14
	*PMP2*	9.79		*SNAI2*	2.51
	*PTGDS*	137.17		*TPH1*	9.32
	*RBP4A*	5.16		*ITGA6*	0.36
	*RBP5*	2.52		*PTGS2*	0.43
	*RBP7*	5.37		*ZBTB7C*	0.40
	*SLC27A1*	3.32			
	*FADS6*	5.95	cholesterol metabolism (10/12)	*ABCA1*	6.99
	*CD36*	3.46		*ABCG1*	13.89
	*ANGPTL4*	11.38		*APOA1*	58.55
	*ACSBG2*	2.14		*APOA4*	5.49
	*ACSL5*	2.89		*CH25H*	2.22
	*SLC45A3*	0.33		*DISP3*	2.28
	*ACSL6*	0.27		*LEPR*	5.05
	*SCD*	0.45		*SOAT1*	2.03
				*CYP17A1*	5.33
fat cell differentiation (10/13)	*ADIPOQ*	4.13		*CYP7A1*	12.82
	*CCDC3*	5.71		*INSIG1*	0.49
	*HTR2A*	2.38		*NSDHL*	0.46

The first number in the parentheses represents the upregulated genes, and the second represents the total genes involved in the term

**Fig. 4:**
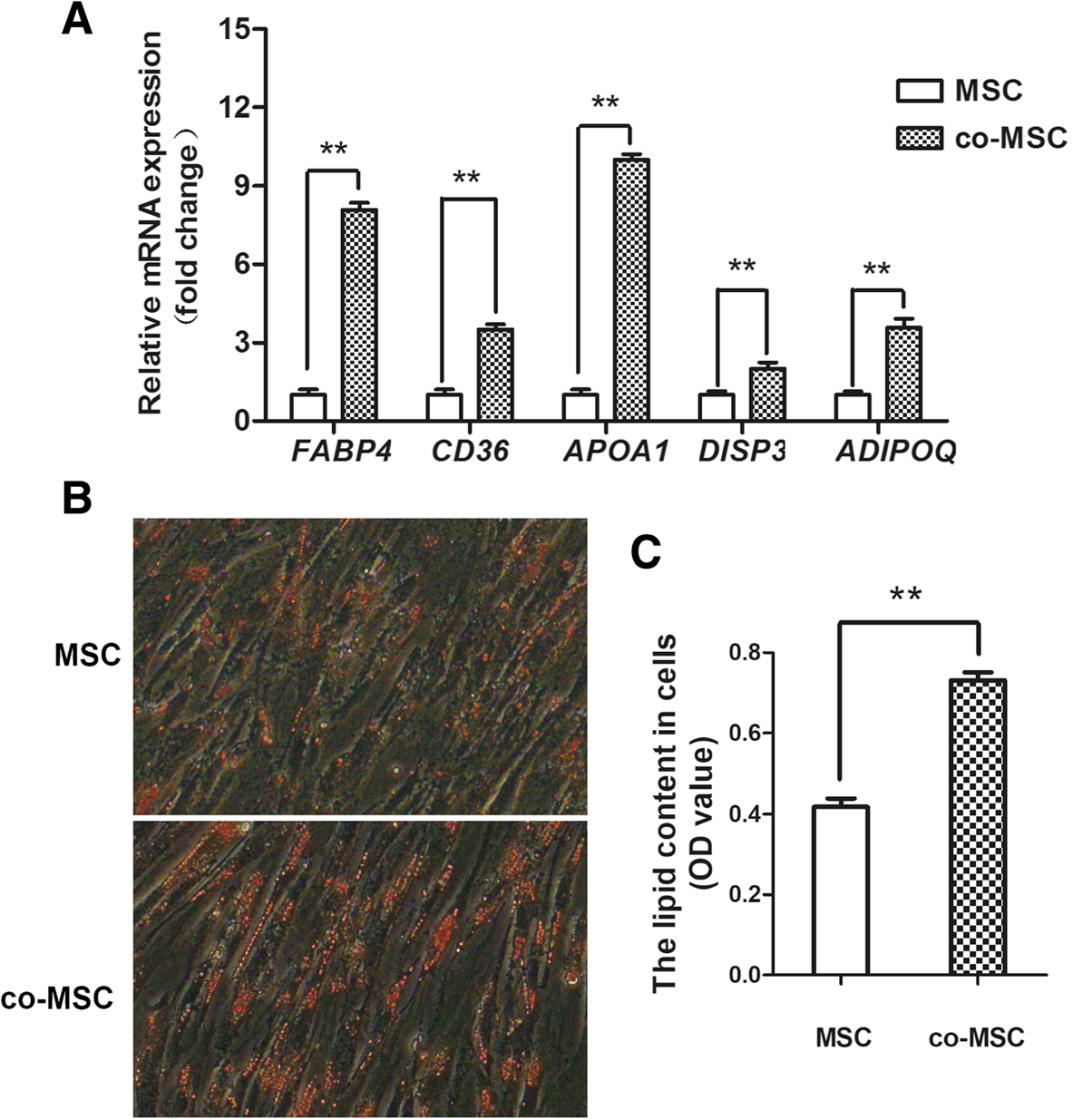
Lipid deposition increased in MSC co-cultured with IMPA. **a** qPCR assays of transcripts of 5 key genes (*FABP4, CD36, APOA1, DISP3,* and *ADIPOQ*) related to lipid deposition of co-cultured or mono-cultured MSC on day 2. These genes were all upregulated in co-cultured MSC. The data are expressed as the mean ± SD (** *P* < 0.01). **b** Oil Red O staining showed that co-cultured MSC contained more lipid droplets on day 4. **c** OD_510_ values of Oil Red O uptake by co-cultured and mono-cultured MSC on day 4. The data are expressed as the mean ± SD (** *P* < 0.01)

This influence of IMPA on lipid deposition in MSC was examined by qPCR of transcripts of 5 key genes involved in lipid deposition in MSC that were co-cultured for 2 days. The transcript abundance of *FABP4*, *CD36*, *APOA1*, *DISP3*, and *ADIPOQ* was all significantly upregulated in co-cultured MSC on day 2 (Fig. 4a). Staining with Oil Red O on day 4 demonstrated that there were more lipid droplets in co-cultured MSC, which further confirmed that IMPA promoted lipid deposition in MSC (Fig. 4b).

### Pathways related to differentiation and lipid deposition in MSC

As differentiation of muscle cells and their lipid deposition are complex processes, the underlying molecular mechanisms may involve interactions between multiple signaling pathways.

**Fig. 5:**
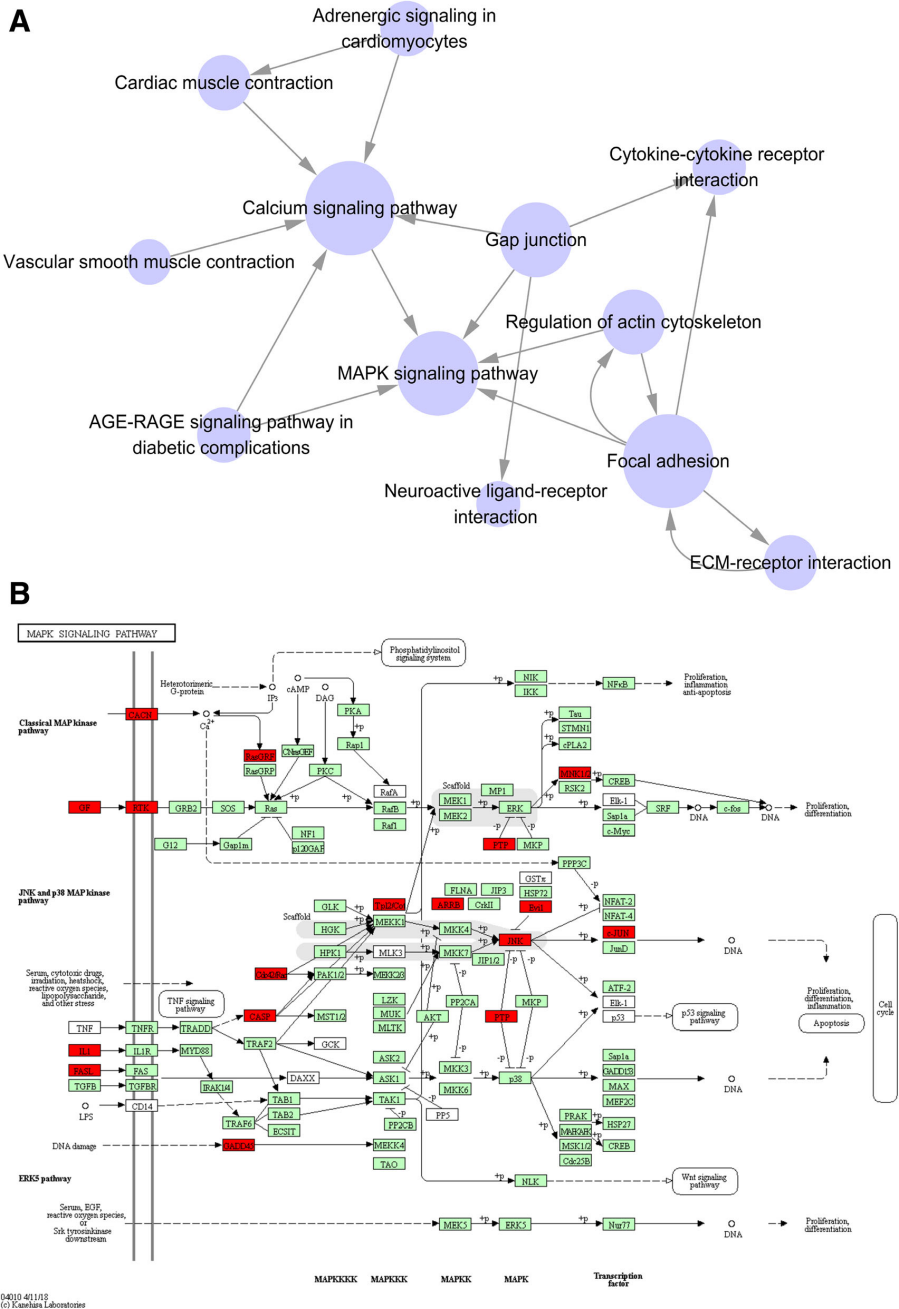
Pathway network and MAPK signaling. **a** The pathway network of interconnected signals was constructed based on the KEGG pathway database and literature. The calcium signaling pathway and MAPK signaling pathway were the two most interactive pathways. The calcium signaling pathway tightly interacted with pathways related to muscle contraction, and MAPK signaling interplayed with cell junction-related pathways. **b** The MAPK signaling pathway was enriched in the present study. The DEG are highlighted in red. The MAPK signaling pathway contains 4 distinct cascades, ERK1/2, JNK/MAPK, p38-MAPK, and ERK5. Few DEG were enriched in ERK1/2 signaling, but several DEG were enriched in JNK signaling

The pathway network of interconnected signals was constructed on the basis of the KEGG pathway database. As shown in Fig. 5a, the calcium signaling pathway and MAPK signaling pathway were the two most interactive pathways. Calcium signaling tightly interacted with pathways related to muscle contraction, and MAPK signaling interplayed with cell junction related pathways. The MAPK signaling pathway functioned downstream of calcium signaling. Thus, MAPK signaling may be the key pathway involved in the present study.

The MAPK signaling pathway contains 4 distinct cascades, ERK1/2, JNK/MAPK, p38-MAPK and ERK5, all of which have been demonstrated to be involved in skeletal myogenesis [22]. The data obtained here indicate that DEG were seldom enriched in the p38-MAPK and ERK5 pathways, and just a few DEG were enriched in the ERK1/2 pathway (Fig. 5b). In contrast, many DEG were enriched in JNK/MAPK signaling, including *Rac2, MAP3K8, ARR3,* the core effector JNK (*MAPK10*), and its downstream effector c-JUN (*JUN*), all indicating that the JNK/MAPK signaling pathway may be the key pathway influencing differentiation of MSC.

Apart from muscle differentiation, MAPK signaling is also involved in lipid metabolism [23, 24]. PPAR signaling occurred downstream of MAPK signaling, and numerous critical genes including *CD36*, *FABP4*, *ADIPOQ*, *APOA1, CYP7A1*, *PLIN2,* and *SLC27A1* were enriched in this pathway, consistent with PPAR signaling being the key pathway regulating lipid deposition in MSC.

Pathways related to cell junctions (ECM-receptor interaction, focal adhesion, regulation of actin cytoskeleton, and gap junction) might form a regulatory network with the MAPK signaling pathway and contribute to the differentiation of MSC and their lipid deposition.

## Discussion

Chickens are very important food animals worldwide and, because all embryonic stages are readily available, they have been widely used as a model for muscle development studies [25]. The skeletal muscle mass is a crucial trait for the economic yield of chickens, and in turn, is largely dependent on the proliferation and differentiation of MSC [3]. This study is the first to investigate the effects of IMPA on MSC in chickens and unveil the molecular mechanisms by transcriptome analysis. The experimental model used MSC, cultured alone or co-cultured so that the influence of IMPA on the MSC could be determined.

The repeatability within and differences between samples of mono-cultured or co-cultured MSC were examined by PCA and hierarchical clustering. There was good repeatability among the replicate samples of each culture treatment and distinction between mono-culture and co-culture. The RNA-seq results for 20 transcripts were well-supported by the qPCR data.

Based on GO terms, 48 and 44 of the 1653 known DEG found here were related to muscle development and lipid deposition. Most of the DEG related to muscle development were downregulated in co-cultured MSC, and DEG related to lipid deposition were upregulated. Immunofluorescence of MHC supported IMPA inhibiting differentiation of MSC, and Oil Red O staining showed concurrent promotion of lipid deposition. This duality of action has not been previously described.

Muscle cell differentiation includes cell migration, cell alignment, and cell fusion and can be expected to result from complex pathway interactions [26, 27]. Muscle cell differentiation occurs under the control of several factors such as myogenic regulatory factors (MRFs) and Pax7 [28–30]. MRFs, including MyoD, Myf5, MyoG, and MRF6, have critical roles in skeletal muscle cell commitment and differentiation [31, 32]. MyoD functions in commitment to satellite cell activation, proliferation, and differentiation [33]. MyoG has a crucial role in the terminal differentiation of muscle cells [32]. Pax7 belongs to the paired box transcription factor family, which can regulate the developmental processes of embryonic myoblasts [34], and Pax7 activates myogenic regulatory factors MyoD and Myf5. In addition, myomaker (TMEM8C), a newly discovered muscle-specific transmembrane protein, is directly involved in cell fusion and it stimulates myoblast fusion in chickens and mammals [35–38]. In the present study, transcripts of *MYOD1*, *MYOG*, *PAX7*, and *TMEM8C* were all significantly reduced in co-cultured MSC, which is consistent with the reduced numbers of MHC-positive cells. These genes, therefore, may be the key genes mediating the effect of IMPA on differentiation of the MSC.

**Fig. 6:**
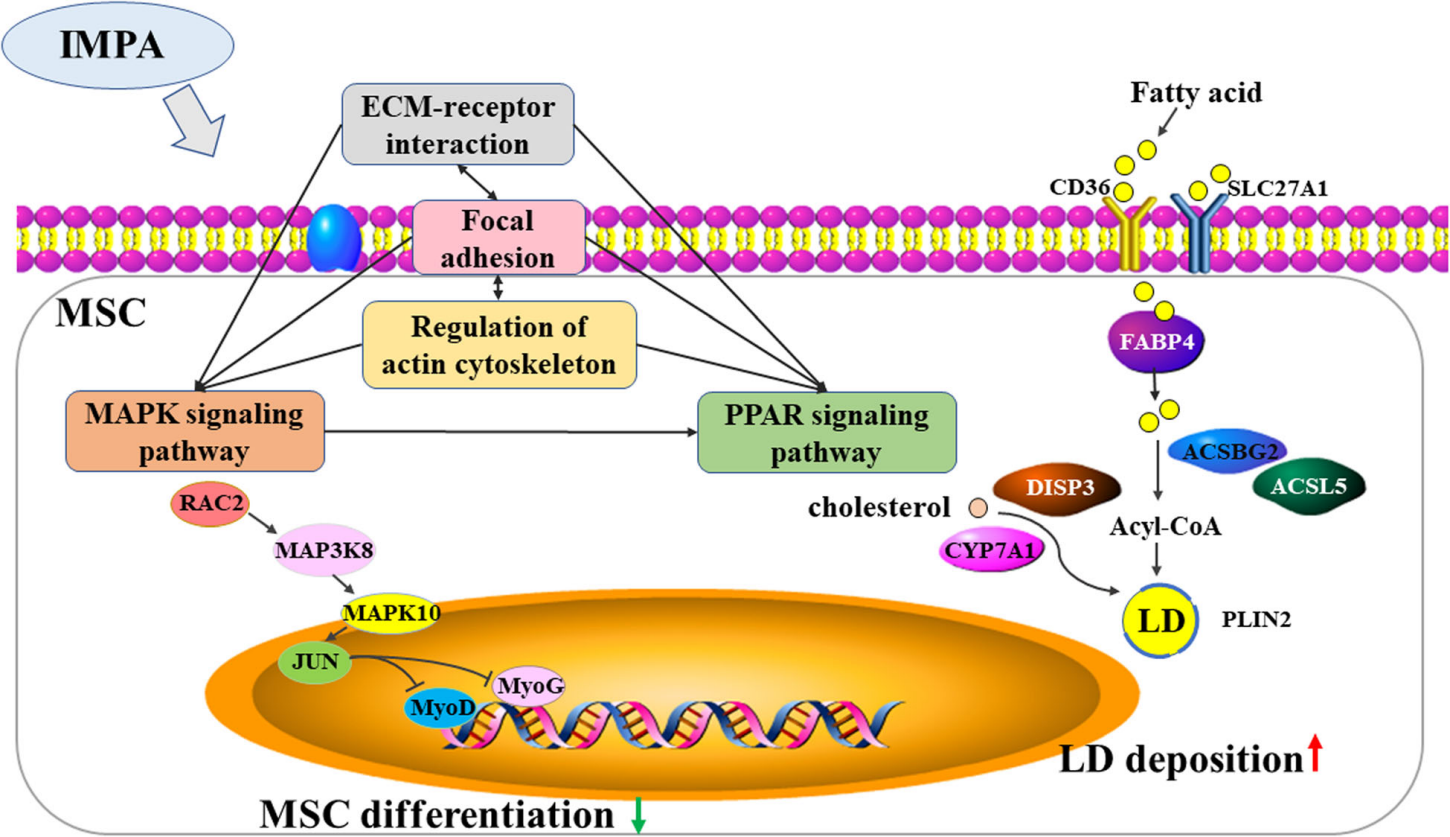
The proposed molecular mechanisms underlying the effects of IMPA on differentiation and lipid deposition in chicken MSC. (1) IMPA suppressed differentiation of MSC via JNK/MAPK signaling: IMPA trigger the upstream kinase Rac2, which subsequently activates MAP3K8, leading to activation of JNK. The activated JNK next activates its downstream effector c-JUN, and subsequently inhibits MyoD and MyoG. Finally, the downregulated MyoD and MyoG inhibit differentiation of MSC. (2) IMPA promote lipid deposition in MSC mainly via PPAR signaling: IMPA influences fatty acid transport in MSC via CD36, SLC27A1, and FABP4, activate fatty acids via ACSBG2 and ACSL5, facilitate cellular cholesterol formation via DISP3 and CYP7A1, and contribute to lipid droplets (LD) via PLIN2. Pathways related to cell junctions are closely interrelated with each other, which form a regulatory network with MAPK signaling and contribute to differentiation and lipid deposition of MSC

It appeared that MAPK signaling was central to the pathway network whereby IMPA influenced the MSC. Many key DEG were enriched in JNK/MAPK signaling. The present findings are consistent with a recent study suggesting that JNK/MAPK signaling inhibits skeletal muscle differentiation by negatively regulating MyoD [39]. The proposed molecular regulatory mechanism by which co-culture with IMPA affected MSC is given in Fig. 6. Signaling via JNK/MAPK is mediated initially by IMPA triggering the upstream kinase Rac2 and subsequently activating MAP3K8, leading to activation of MAPK10. The activated MAPK10 next activates its downstream effector JUN, and transcriptional factors MyoD and MyoG were further downregulated. Finally, the downregulated MyoD and MyoG inhibited differentiation of MSC.

The formation of lipid droplets is a highly conserved process that involves the activation of fatty acids, synthesis of neutral lipid, and the formation of droplets, involving the products of many genes and pathway interactions [40]. CD36 and SLC27A1 are both fatty acid transport proteins involved in the translocation of long-chain fatty acids [41, 42]. FABP4, fatty acid binding protein 4, plays a role in intracellular lipid transport [43]. ACSBG2 and ACSL5 are both acyl-CoA synthetases, playing a critical role in the activation of fatty acids via esterification with coenzyme-A [44]. In addition, DISP3 and CYP7A1 play a role in the accumulation of cellular cholesterol and are involved in droplet formation [45]. PLIN2, located on the droplet surface, is the predominant perilipin in skeletal muscle and is upregulated during droplet formation, forming a protective coat inhibiting lipolysis [46–48]. In the present study, *CD36*, *SLC27A1*, *FABP4*, *ACSBG2*, *ACSL5*, *DISP3*, *CYP7A1*, and *PLIN2* were all upregulated in co-cultured MSC, consistent with their increased numbers of lipid droplets. Thus, these 8 genes might be key genes involved in the effect of IMPA on lipid deposition in MSC (Fig. 6).

Of these 8 genes, 7 were involved in PPAR signaling, indicating that it might be the key pathway for lipid deposition in MSC, as reflected in the observations from Oil Red O staining. In addition to the proposed role of MAPK signaling in muscle differentiation, it is also involved in lipid metabolism [49, 50]. Several studies have shown that MAPK signaling could modulate PPAR [51, 52], consistent with the present conclusion that this mediated the effect of IMPA in promoting lipid deposition in MSC.

Several pathways related to cell junctions were also enriched in co-cultured MSC, including ECM-receptor interaction, Focal adhesion, CAMs, Gap junction, Cytokine-cytokine receptor interaction and Regulation of actin cytoskeleton, all of which were closely interrelated. Focal adhesion is a cluster of integrin transmembrane receptors that mediate dynamic interactions between the extracellular matrix and the actin cytoskeleton [53, 54]. In the present study, a series of genes encoding integrins (*ITGA4*, *ITGA6*, *ITGA8*, *ITGA9*, *ITGAV* and *ITGB8*) were differentially expressed. These genes simultaneously participated in 4 pathways (ECM-receptor interaction, focal adhesion, CAMs, and regulation of actin cytoskeleton). In addition, *PDGFRA* and *PDGFRB* encoded proteins correlated with Focal adhesion, Gap junction, Cytokine-cytokine receptor interaction, and Regulation of actin cytoskeleton.

These cell junction-related pathways broadly participate in muscle differentiation [55–57]. During the process of differentiation of MSC, significant changes take place in cell morphology, ECM components, and actin cytoskeleton remodeling. [58]. In addition, several studies suggest that cell junction-related pathways are also involved in lipid deposition [52, 59, 60].

Several of the DEG identified here in co-cultured versus mono-cultured MSC (*FGF12*, *FGF16*, *FGF19* and *FGF9*) belonging to the FGF family were reflected in MAPK signaling and also participated in the regulation of the actin cytoskeleton. It seems likely that pathways related to cell junctions may form a regulatory network with MAPK signaling and contribute to differentiation and lipid deposition in MSC.

We have developed a novel co-culture system designed to mimic the physiologic interactions between skeletal muscle cell and intramuscular preadipocytes. Our results demonstrated that intramuscular preadipocytes inhibited muscle cell differentiation and promoted lipid deposition. A deep understanding of cross-talk between muscle cells and intramuscular adipocytes could be utilized to improve meat quality in chickens. In humans, excessive intracellular lipid accumulation in skeletal muscle is associated with some metabolic diseases such as insulin resistance and type 2 diabetes [61–63]. The data suggest that some factor secreted by adipocytes induced intracellular lipid accumulation in muscle cells, which provide important insights for livestock meat quality and human metabolic disease.

## Conclusions

This study with chicken cells showed that IMPA impeded differentiation of MSC while promoting their lipid deposition. KEGG pathway analysis indicated that IMPA might inhibit differentiation via the JNK/MAPK pathway, and promote lipid deposition via the PPAR pathway. Pathways related to cell junctions may also contribute to differentiation and lipid deposition in MSC. Although much experimentation is required to develop these predictions, they provide new clues for exposing the molecular mechanisms underlying the interplay between skeletal muscle and intramuscular fat in chickens.

## Additional files

Additional file 1: **Table S1.** The specific primers for qPCR in this study. (XLSX 12 kb)

Additional file 2: **Table S2.** The screened DEG in the study. (XLSX 218 kb)

Additional file 3: **Table S3**. The enriched GO-terms based on 1653 DEG. (XLSX 19 kb)

Additional file 4: **Table S4.** The DEG related to myogenic differentiation and muscle development. (XLSX 14 kb)

Additional file 5: **Table S5.** The DEG related to lipid deposition. (XLSX 14 kb)

Additional file 6: **Table S6.** The enriched pathways based on 1653 DEG. (XLSX 16 kb)

## Abbreviations

ACSBG2: Long chain fatty acid CoA ligase
ADIPOQ: Adiponectin
APOA1: Apolipoprotein A1
ARR3: Arrestin 3
BP: Biological Process
CAMs: Cell adhesion molecules
CD36: CD36 molecule
CYP7A1: Cholesterol 7 alpha monooxygenase
DEG: Differentially expressed genes
DISP3: Dispatched RND transporter family member 3
DMEM: Dulbecco’s modified Eagle’s medium
ECM: Extracellular matrix
ERK1/2: The extracellular signal- related kinases
FABP4: Fatty acid binding protein 4
FGF12: Fibroblast growth factor 12
FGF16: Fibroblast growth factor 16
FGF19: Fibroblast growth factor 19
FGF9: Fibroblast growth factor 9
FNDC5: Fibronectin type III domain containing 5
GO: Gene Ontology
HOMER1: Homer scaffolding protein1
IGF2: Insulin like growth factor 2
IMPA: Intramuscular preadipocytes
ITGA4: Integrin subunit alpha 4
ITGA6: Integrin subunit alpha 6
ITGA8: Integrin subunit alpha 8
ITGA9: Integrin subunit alpha 9
ITGAV: Integrin alpha-V
ITGB8: Integrin beta 8
JNK: C-Jun N-terminal kinases
JUN: Transcription factor AP-1
KEGG: Kyoto Encyclopedia of Genes and Genomes
LD: Lipid droplet
MAPK: Mitogen-activated protein kinase
MF: Molecular Function
MHC: Myosin heavy chain
MRFs: Myogenic regulatory factors
MSC: Muscle satellite cells
Myf5: Myogenic factor 5
MYF6: Myogenic factor 6
MYH10: Myosin Heavy Chain10
MYH13: Myosin Heavy Chain 13
MYH15: Myosin Heavy Chain 15
MYH1E: Myosin Heavy Chain 1
MYL1: Myosin Light Chain 1
MYL4: Myosin Light Chain 4
MYO16: Myosin XVI
MyoD: Myogenic differentiation
MyoG: Myogenin
NTN3: Netrin1
PCA: Principal component analysis
PDGFRA: Platelet-derived growth factor receptor alpha
PDGFRB: Platelet-derived growth factor receptor beta
PLIN2: Perilipin 2
PPAR: Peroxisome proliferators-activated receptors
Rac2: Rac family small GTPase 2
SLC27A1: Long-chain fatty acid transport protein 1
TMEM8C: Transmembrane protein 8C
